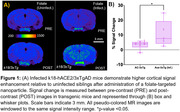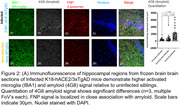# In Vivo Characterization of Neuroinflammation Using a Folate Receptor Targeted Nanoparticle Platform in a Mouse Model of SARS‐CoV‐2 Infection and Alzheimer’s Disease

**DOI:** 10.1002/alz.089974

**Published:** 2025-01-09

**Authors:** Parag A Parekh, Andrew A Badachhape, Lauren Bonilla, Alexander R Kneubehl, Renuka Menon, Jennifer L Clinton, Prajwal Bhandari, Rohan Bhavane, Prasad Admane, Mayank Srivastava, Shannon E Ronca, Eric Tanifum, Ananth Annapragada, Ketan Ghaghada

**Affiliations:** ^1^ Baylor College of Medicine, Houston, TX USA; ^2^ Texas Children's Hospital, Houston, TX USA; ^3^ Texas Children's Hospital/Baylor College of Medicine, Houston, TX USA

## Abstract

**Background:**

Clinical studies in COVID‐19 patients have demonstrated evidence of neuroinflammation in patients infected with SARS‐CoV‐2. In this pre‐clinical work, we investigate neuroinflammation in vivo in a mouse model of Alzheimer’s disease (AD) that exhibits both amyloid and tau pathology, the 3xTg‐AD mice crossed with K18 hAce2 and infected with SARS‐CoV‐2 using a folate receptor‐targeted nanoprobe for molecular MRI (mMRI) followed by ex vivo histopathology.

**Method:**

In vivo studies with SARS‐CoV‐2 were performed in an animal biosafety level 3 (ABSL3) facility. 3xTg transgenic mice, a model of amyloid and tau pathology, were bred with k18‐hACE2 transgenic mice to generate double transgenic k18‐hACE2/3xTg transgenic mice that express hACE2 receptors (critical for viral infection) and develop amyloid and tau pathology. 11–14‐month‐old transgenic k18‐hACE2/3xTg mice (n=6) were infected with 10^3^ PFU of SARS‐CoV‐2 Delta variant and underwent mMRI studies 10 days post‐infection. Age‐matched non‐infected k18‐hACE2/3xTg mice (n=6) were used as controls. All mice underwent pre‐contrast and post‐contrast T1‐weighted MRI (T1w‐MRI). Post‐contrast T1w‐MRI was performed 4 days after systemic administration of a high T1 relaxivity folate targeted liposomal‐Gd contrast agent. Signal changes between pre‐contrast and post‐contrast MRI were determined in target regions of the brain. Animals were euthanized after final imaging session for microscopic analysis of the brain.

**Result:**

Non‐infected K18‐hACE2/3xTg mice showed relatively low brain signal enhancement in post‐contrast MRI (3.3 %) consistent with the absence of frank neuroinflammation. In contrast, K18‐hACE2/3xTg transgenic mice infected with SARS‐CoV‐2 demonstrated significantly higher brain signal enhancement (+10.9%) in post‐contrast T1w‐MRI compared to pre‐contrast MRI (Figure 1) suggesting infection‐induced neuroinflammation. Signal enhancement was seen in cortical and hippocampal regions of the brain. Microscopic analysis of mice brain sections depicting the hippocampus region show the folate nanoparticles in the vicinity of amyloid deposits. Quantitative analysis shows increasing amyloid deposition in infected mice. Activated microglia in infected mice staining is also observed at higher levels.

**Conclusion:**

Data suggests that the hybrid hACE2‐3xTg‐AD mice exhibit neuroinflammation and increased amyloid deposition following SARS‐CoV2 infection indicating acceleration of AD pathology in these mice.